# Case Report: A Case Report of a Histological Transformation of *ALK*-Rearranged Adenocarcinoma With High Expression of PD-L1 to Squamous Cell Carcinoma After Treatment With Alectinib

**DOI:** 10.3389/pore.2021.637745

**Published:** 2021-04-01

**Authors:** Yan Zhang, Yaping Qin, Hongen Xu, Qihui Yao, Yalan Gao, Yushu Feng, Jingli Ren

**Affiliations:** ^1^ Department of Pathology, The Second Affiliated Hospital of Zhengzhou University, Zhengzhou, China; ^2^ Department of Precision Medicine Center, The Second Affiliated Hospital of Zhengzhou University, Zhengzhou, China

**Keywords:** ALK-Rearranged adenocarcinoma, PD-L1, squamous cell carcinoma, alectinib, histological transformation

## Abstract

We report an anaplastic lymphoma kinase (ALK)-positive patient shows a poor response to the ALK inhibitor alectinib due to the high expression of programmed death-ligand 1 (PD-L1). After treatment with alectinib, the pathological form changed from adenocarcinoma into squamous cell carcinoma without novel genetic changes. This case may reveal a direct relationship between ALK mutation and a high level of PD-L1 expression.

## Background

Non-small cell lung cancer (NSCLC) with anaplastic lymphoma kinase (*ALK*) gene rearrangement is a distinct subtype of lung cancer, and it is highly sensitive to the *ALK* kinase inhibitors such as crizotinib or alectinib [1, 2]. The *ALK* fusion mutation is called the “diamond mutation”, and the patient who has a tumor harbouring *ALK* mutation usually has a more prolonged progression-free survival (PFS). For *ALK*-positive patients taking crizotinib, the median PFS was 11.8 months [3], and 62% of patients can achieve a 3-years PFS after treatment with alectinib [4]. Drug resistance frequently occurs due to alterations in the oncogene-driven genes and phenotypic transformation [3]. Various resistance mechanisms, similar to other oncogene-driven cancers treated with tyrosine kinase inhibitors (TKIs), may be classified into three different groups: 1) alterations in the oncogene-driven genes (such as *ALK* L1196M or C1156Y mutations [5]); 2) activation of bypass pathways (for example insulin-like growth factor 1 receptor (*IGF-1R*) [6], SRC proto-oncogene, non-receptor tyrosine kinase (*SRC*) [7] and mitogen-activated protein/extracellular signal-regulated kinase (ERK) kinase (*MEK*/*ERK*) [8] activation); 3) phenotypic transformation (to small cell lung cancer) [9]. The histological shift has been reported as a resistance mechanism in 3–14% of the patients treated with epidermal growth factor receptor (*EGFR*) TKIs [10, 11]. However, phenotypic transformation is rarely reported in *ALK*-positive patients. Herein, we presented a case of *ALK* rearrangement-positive adenocarcinoma with high expression of programmed death-ligand 1 (PD-L1) that was transformed into squamous cell carcinoma after administration of alectinib.

### Case Presentation

**FIGURE 1:**
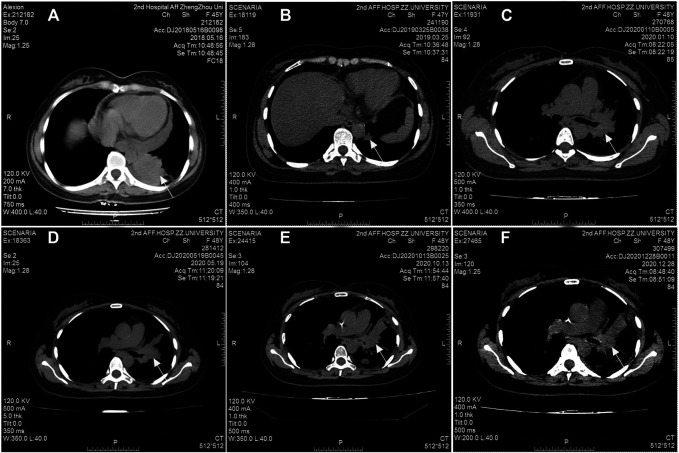
Radiographic response to treatment. Computed tomography (CT) scans at initial diagnosis **(A)**, seven months after taking alectinib for treatment **(B)**, 17 months after taking alectinib for treatment **(C)**, and two months after taking ceritinib for treatment **(D)**, before the second chemotherapy cycle **(E)**, before the 5th chemotherapy cycle **(F)**.

**FIGURE 2:**
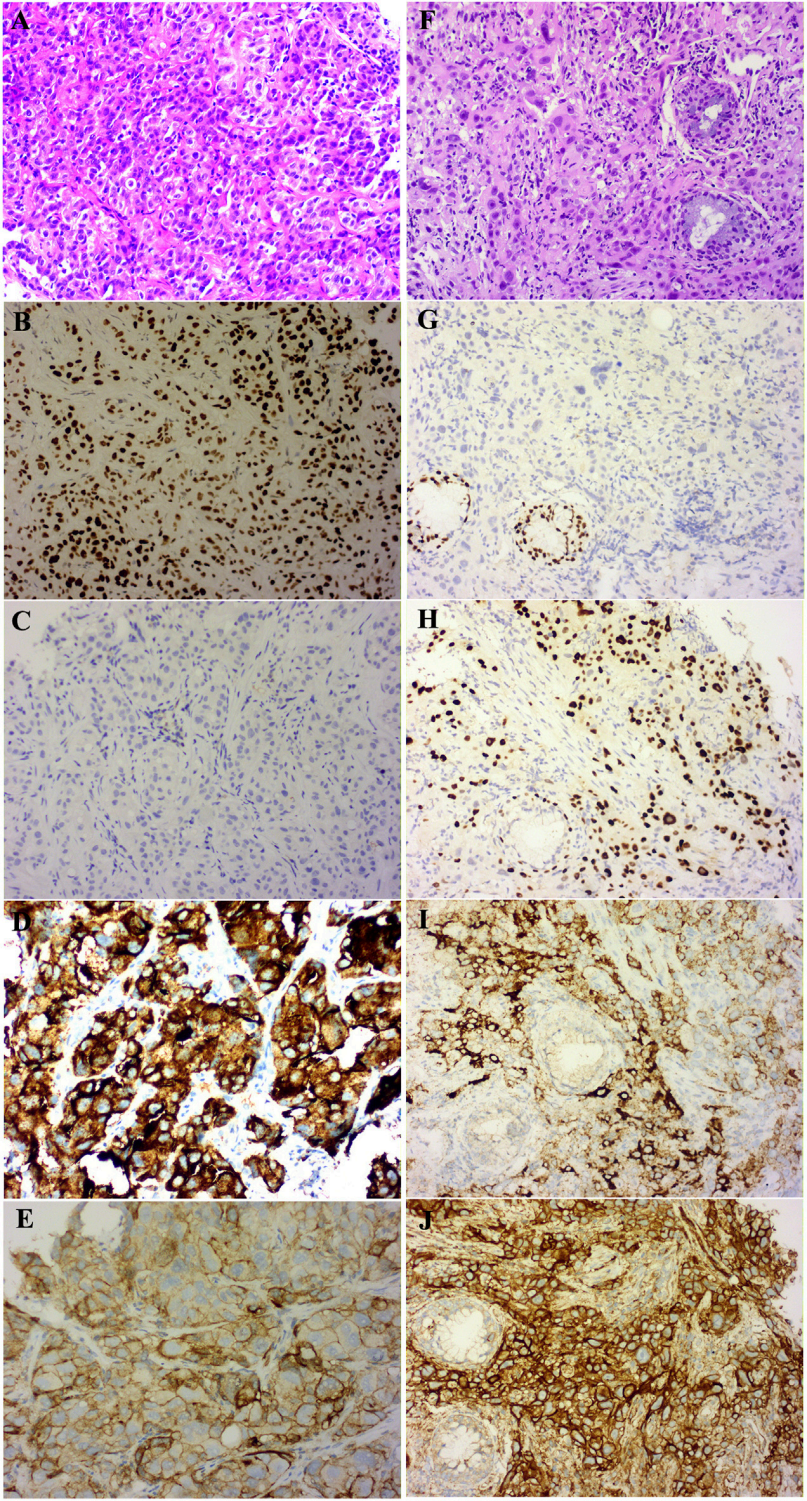
Pathology with Hematoxylin-eosin (HE) stained and immunohistochemistry (IHC) results of the patient (×20 magnification). The baseline adenocarcinoma (ADC) HE result **(A)**, ADC sample IHC analysis shows TTF-1—positive **(B)**, p40—negative **(C)**, ALK (clone D5F3)—positive **(D)** and PD-L1 (clone SP263)—TPS of 80% **(E)**; the transformed squamous cell carcinoma (SCC) HE result **(F)**, SCC sample IHC analysis shows TTF-1—negative **(G)**, p40—positive **(H)**, ALK (clone D5F3)-positive **(I)** and PD-L1 (clone SP263)—TPS of 85% **(J)**.

**FIGURE 3:**
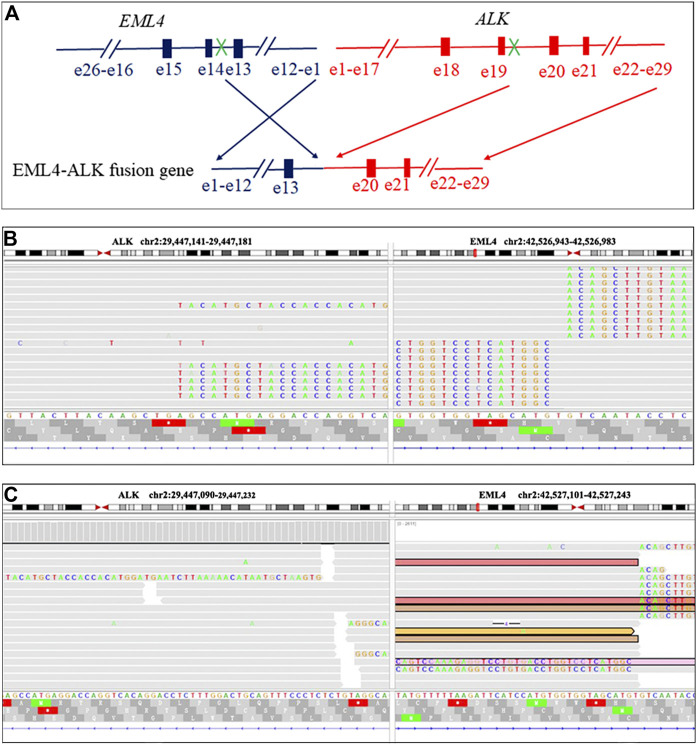
EML4-ALK fusion in the present case visualized using the Integrative Genomics Viewer (IGV). Diagrammatic sketch of EML4-ALK fusion result **(A)**. The IGV display of EML4-ALK fusion in an adenocarcinoma specimen of EML4-ALK fusion results **(B)**. The IGV display of EML4-ALK fusion in a squamous cell carcinoma specimen of EML4-ALK fusion results **(C)**.

A 47-year-old female, who is a never-smoker, had an intermittent dry cough with back pain without obvious inducement, continued without remission in May 2018. Chest computed tomography (CT) showed a mass (about 42 × 55 mm) in his left lower lobe (Figure 1A) accompanied by the liver, scapula, and right frontal lobe metastases. The pathological examination indicated that his liver metastasis was a poorly differentiated adenocarcinoma. The immunohistochemical results (Figure 2) showed that thyroid transcription factor-1 (TTF-1), Napsin A, and ALK (D5F3, Ventana Medical Systems, Inc.; other antibodies are from Beijing Zhongshan Golden Bridge Co., Ltd.) were positive, while p40, CK5/6, and CK7 were negative. An *EML4*-*ALK* fusion was detected by a mutational analysis using an amplification refractory mutation system-PCR. The patient was prescribed crizotinib on June 1st, 2018. The liver and heart function tests on July 26th, 2018 showed that ALT was 1079 U/L (reference range 5–40 U/L), and CK-MB was 250 U/L (reference range <24 U/L), which were significantly higher than normal. Since the previous liver and heart functions of the patient were normal, the abnormality of the examination results was assumed as an adverse reaction to the drug. The patient was switched to alectinib for targeted therapy on August 10, 2018. The function of the liver and heart from the patient were tested in a month-treatment. It is found that the ALT was back to normal (23 U/L), CK-MB was 54 U/L that is close to the normal value, and no other adverse reactions were observed. On March 25th, 2019, we performed the chest CT on the patient to reexamine the tumor condition and found it almost disappeared (Figure 1B). The patient continued taking alectinib for maintenance treatment. On January 10th, 2020, a chest CT reexamination showed soft tissue density display in the left hilum (Figure 1C). The tumor recurrence was considered, and subsequently the patient developed an intermittent cough that gradually worsened. Considering the disease progression, the clinician recommended ceritinib for targeted therapy. On May 19th, 2020, the chest CT scan identified tumors in his left hilum (Figure 1D) and lower left lung, and the tumors blocked the left main bronchus. The bronchoscope could not pass through the bronchus. The biopsy pathology indicated a poorly differentiated squamous cell carcinoma. The immunohistochemical results (Figure 2) showed that TTF-1, Napsin A, Chromogranin A (CgA), Synaptophysin (Syn), and CD56 were negative, while P40, P63, *Pan* Keratin (AE1/AE3), and ALK (D5F3) were positive. The squamous cell carcinoma and adenocarcinoma samples were examined by next-generation sequencing (NGS) of 547 tumor-related genes (NanOncoPanel v1.1, Nanodigmbio (Nanjing) Biotechnology Co., Ltd.). Covering a 2.05 Mb region of the human genome, NanOncoPanel v1.1 is an NGS-based assay for detecting substitutions, insertions, and deletion (indels), copy number alterations (CNAs), and genomic rearrangements in 547 cancer-relevant genes. The NGS results (Figure 3) of squamous cell carcinoma and adenocarcinoma samples revealed the fusion of *EML4* Exon 13 and *ALK* Exon 20 without other pathogenic mutations detected. Anlotinib was given on June 8th, 2020, and the expression level of PD-L1 in two specimens was tested. Both specimens tumors had a PD-L1 (Ventana Medical Systems, Inc.) TPS of 80% and 85%, as determined with the SP263 assay (Figure 2 E&J). The patient began to receive abusartan combined with carboplatin chemotherapy on September 9th, 2020, and had completed five cycles of chemotherapy as of December 31st. Combined with the CT results (Figure 1E&F), the patient's condition was assessed as stable disease. This study was approved by the Ethics Committee of the Second Affiliated Hospital of Zhengzhou University, and in accordance with the guidelines of the Declaration of Helsinki, all participating individuals signed informed consent.

## Discussion

To the best of our knowledge, this is the first clinical case demonstrating the relationship between histological morphological changes after administration of alectinib and the expression of PD-L1. Although histological transformation has been reported in the patients treated with EGFR-TKIs, there are very few reports of this transformation in *ALK*-positive patients, especially the transformation of adenocarcinoma into squamous cell carcinoma. Given that our patient is a never-smoker, *EML4* Exon 13 and *ALK* Exon 20 fusion mutations were detected in the two biopsies at first diagnosis and recurrence, and 80% of the tumor cells in the two specimens showed diffuse and strong positive expression of PD-L1, the tissues after resistance were not likely to originate from an independent new tumor. In addition, primary *ALK*-positive squamous cell carcinoma often showed a remarkably positive response to alectinib [12, 13], but in this case the squamous cell carcinoma was not sensitive to alectinib. Since the patient was taking alectinib for treatment without chemotherapy or radiotherapy, we believe that changes in the initial tumor morphology could be caused by treatment with alectinib. The mechanism can be partly explained by the fact that the histological changes caused by alectinib, which is a highly selective *ALK*-TKI, inhibit multiple crizotinib-resistant *ALK* mutations. Importantly, the IC50 of inhibited cell growth was 12 nmol/L, which was about one-sixth of crizotinib [9]. These characteristics of alectinib put more severe selective pressure on the adenocarcinoma cells during treatment with alectinib. After overcoming the evolutionary process of selective pressure, alectinib-resistant tumor cells undergo biological changes more frequently. This may explain the appearance of recurring tumors within ten months of continuing alectinb therapy after the primary tumor almost completely disappeared in this case.

Patients with *ALK* fusions usually have a longer PFS. Nevertheless, in this case, the patient was taking alectinib for treatment, and the PFS was only 18 months. The most likely explanation is that the patient has a high level (PD-L1 TPS of ≥50%) of PD-L1 expression. We reviewed the first liver puncture specimens and found that the tumors had a PD-L1 TPS of 80%. Yang et al. [3] revealed that patients with *ALK*-positive lung adenocarcinoma followed by strong PD-L1 expression have a shorter PFS than those with negative PD-L1 expression after crizotinib treatment. Hsu et al. [14] found similar results among patients with *EGFR*-mutant lung adenocarcinoma treated with EGFR-TKIs. Therefore, the shorter PFS in our patient can be explained by alectinib therapy.

Our study provides evidence that 1) although crizotinib is the first-line medication for the treatment of ALK-positive patients, the serious abnormality occuring on liver and heart needs more attention after giving crizotinib. 2) alectinib has been proved a better curative effect for the ALK-rearrangement lung adenocarcinoma, and repeated biopsies are needed to determine histological changes when resistance emerges. 3) Patients having a high level of PD-L1 expression are more likely to have drug resistance. For clinicians, a patient who shows a PD-L1 TPS of ≥50% will need more careful monitoring of drug resistance during treatment, thus the appropriate and timely therapy can be performed to improve the survival of patients.

## Data Availability

The original contributions presented in the study are included in the article/Supplementary Material, further inquiries can be directed to the corresponding author.
